# Supplementary material for: Prevalence of Gastrointestinal Symptoms in Chinese Community-Dwelling Adults with and without Diabetes
Source: Nutrients. 2022 Aug 26;14(17):3506. doi: 10.3390/nu14173506 (PMC9459935; doi:10.3390/nu14173506)
Supplement: Supplementary file 1 [file nutrients-14-03506-s001.zip › nutrients-1852255-supplementary.pdf]

Supplementary material

Table S1. Comparison of gastrointestinal symptoms in participants with normoglycemia, prediabetes and diabetes without glucose-lowering therapies

	Total (*n* = 1231)	Normoglycemia （*n* = 730）	Prediabetes （*n* = 360）	Diabetes without glucose-lowering therapies （*n* = 141）	*P*
At least one GI symptom, *n* (%)	230 (18.7%)	129 (17.7%)	71 (19.7%)	30 (21.3%)	0.50
Abdominal pain, *n* (%)	15 (1.2%)	9 (1.2%)	4 (1.1%)	2 (1.4%)	0.87
Irritable bowel syndrome, *n* (%)	26 (2.1%)	10 (1.4%)	14 (3.9%)*	2 (1.4%)	0.02
Ulcer-like dyspepsia, *n* (%)	15 (1.2%)	9 (1.2%)	4 (1.1%)	2 (1.4%)	0.87
Early satiety, *n* (%)	26 (2.1%)	14 (1.9%)	10 (2.8%)	2 (1.4%)	0.54
Postprandial fullness, *n* (%)	22 (1.8%)	13 (1.8%)	5 (1.4%)	4 (2.8%)	0.55
Nausea, *n* (%)	7 (0.6%)	4 (0.5%)	3 (0.8%)	0 (0.0%)	0.74
Retching, *n* (%)	2 (0.2%)	1 (0.1%)	1 (0.3%)	0 (0.0%)	0.65
Vomiting, *n* (%)	2 (0.2%)	1 (0.1%)	1 (0.3%)	0 (0.0%)	0.65
Loss of appetite, *n* (%)	12 (1.0%)	8 (1.1%)	2 (0.6%)	2 (1.4%)	0.53
Abdominal fullness or bloating, *n* (%)	36 (2.9%)	21 (2.9%)	11 (3.1%)	4 (2.8%)	0.97
Gastroesophageal reflux symptoms, *n* (%)	35 (2.8%)	22 (3.0%)	8 (2.2%)	5 (3.5%)	0.66
Dysphagia, n (%)	2 (0.2%)	1 (0.1%)	1 (0.3%)	0 (0.0%)	0.65
Diarrhea, *n* (%)	76 (6.2%)	43 (5.9%)	23 (6.4%)	10 (7.1%)	0.85
Constipation, *n* (%)	70 (5.7%)	38 (5.2%)	22 (6.1%)	10 (7.1%)	0.62
Fecal incontinence, *n* (%)	8 (0.6%)	7 (1.0%)	1 (0.3%)	0 (0.0%)	0.43
Symptom complex					
Upper GI symptoms€, *n* (%)	96 (7.8%)	56 (7.7%)	28 (7.8%)	12 (8.5%)	0.94
Lower GI symptoms∲, *n* (%)	147 (11.9%)	83 (11.4%)	44 (12.2%)	20 (14.2%)	0.63

Data are presented as number (%).
€: A combined prevalence of early satiety, postprandial fullness, nausea, retching, vomiting, loss of appetite, abdominal fullness or bloating, gastroesophageal reflux symptoms, and dysphagia.
∲: A combined prevalence of diarrhea, constipation, and fecal incontinence.
The prevalence of GI symptoms across groups was compared using Chi-squared tests.
Logistic regression analyses were used to compare GI symptoms between the groups after adjusting for age and gender.
* $P < 0.05$, compared to subjects with normoglycemia after adjusting for age and gender.

Table S2. The clinical characteristics in diabetes with and without gastrointestinal symptoms

	Without GI symptoms (*n* = 171)	With GI symptoms (*n* = 43)	*P*
Female, *n* (%)	100 (58.5%)	21 (48.8%)	0.25
Age (Y)	57.00 (51.00, 63.00)	60.00 (50.00, 66.00)	0.002
BMI (kg/m²)	25.88 (23.62, 28.44)	26.19 (24.39, 28.03)	0.69
WC (cm)	88.10 (81.20, 95.10)	90.20 (86.20, 97.50)	0.12
SBP (mmHg)	141.00 (127.00, 154.00)	141.00 (127.00, 156.00)	0.85
DBP (mmHg)	85.00 (77.00, 93.00)	85.00 (76.00, 95.00)	0.79
**Smoking status**			0.89
Never, *n* (%)	135 (78.9%)	33 (76.7%)	0.75
Former, *n* (%)	9 (5.3%)	2 (4.7%)	0.87
Current, *n* (%)	27 (15.8%)	8 (18.6%)	0.66
**Drinking status**			0.98
None, *n* (%)	116 (67.8%)	33 (76.7%)	
less than one drink a week, *n* (%)	14 (8.2%)	3（7.0%）	
1-2 drinks a week, *n* (%)	13（7.6%）	2（4.7%）	
3-6 drinks a week, *n* (%)	12（7.0%）	2（4.7%）	
7-10 drinks a week, *n* (%)	2（1.2%）	0（0.0%）	
11-30 drinks a week, *n* (%)	7（4.1%）	1（2.3%）	
More than 30 drinks a week, *n* (%)	7（4.1%）	2（4.7%）	
Duration of diabetes (years)			0.06
＜5 , *n* (%)	127 (74.3%)	39 (90.7%)	
5-10, *n* (%)	22 (12.9%)	3 (7.0%)	
＞10 , *n* (%)	22 (12.9%)	1 (2.3%)	
Glucose-lowering therapies.			0.83
Oral hypoglycemic agents only, *n* (%)	37 (21.6%)	9 (20.9%)	
Insulin ± oral hypoglycemic agents, *n* (%)	11 (6.4%)	1 (2.3%)	
Lifestyle measures only, *n* (%)	111 (64.9%)	30 (69.8%)	
Unclassified, *n* (%)	12 (7.0%)	3 (7.0%)	
Newly diagnosed diabetes, *n* (%)	97 (56.7%)	26 (60.5%)	0.66
FPG (mmol/L)	6.22 (5.71, 7.05)	6.72 (6.24, 7.05)	0.46
HbA1c (%)	6.10 (5.80, 6.50)	6.10 (5.80, 6.40)	0.94
BUN (mmol/L)	5.06 (4.19, 6.09)	5.35 (4.31, 6.48)	0.49
Cr (umol/L)	54.00 (46.00, 64.00)	59.00 (55.00, 65.00)	0.02
TC (mmol/L)	4.73 ± 0.97	4.88 ± 1.08	0.38
TG (mmol/L)	1.58 (1.09, 2.12)	1.43 (0.97, 1.83)	0.22
HDL (mmol/L)	1.39 (1.24, 1.57)	1.35 (1.27, 1.61)	0.62
LDL (mmol/L)	2.64 ± 0.69	2.58 ± 0.80	0.61
Proteinuria, *n* (%)	60 (35.1%)	11 (25.6%)	0.24
Retinopathy, *n* (%)	23 (13.5%)	2 (4.7%)	0.11

Abbreviation：BMI, body mass index; WC,waist circumference; SBP, systolic blood pressure; DBP, diastolic blood pressure; FPG,fasting plasma glucose; HbA1c, glycosylated hemoglobin; BUN, Blood urea nitrogen; Cr, creatinine; TC, total cholesterol; TG, triglyceride; HDL, high density lipoprotein; LDL, low density lipoprotein.

Table S3. Comparison of gastrointestinal symptoms in participants with diabetes under different glucose-lowering therapies

	Lifestyle measures only (*n* = 141)	Oral hypoglycemic agents only (*n* = 46)	Insulin ± oral hypoglycemic agents (*n* = 12)	Unclassified (*n* = 15)	*P*
Total GI symptom, *n* (%)	30 (21.3%)	9 (19.6%)	1 (8.3%)	3 (20.0%)	0.83
Abdominal pain, *n* (%)	2 (1.4%)	0 (0.0%)	0 (0.0%)	0 (0.0%)	1.00
Irritable bowel syndrome, *n* (%)	2 (1.4%)	2 (4.3%)	0 (0.0%)	0 (0.0%)	0.57
Ulcer-like dyspepsia, *n* (%)	2 (1.4%)	0 (0.0%)	0 (0.0%)	0 (0.0%)	1.00
Early satiety, *n* (%)	2 (1.4%)	1 (2.2%)	0 (0.0%)	2 (13.3%)	0.09
Postprandial fullness, *n* (%)	4 (2.8%)	0 (0.0%)	0 (0.0%)	1 (6.7%)	0.38
Loss of appetite, *n* (%)	2 (1.4%)	0 (0.0%)	0 (0.0%)	0 (0.0%)	1.00
Abdominal fullness or bloating, *n* (%)	4 (2.8%)	0 (0.0%)	0 (0.0%)	1 (6.7%)	0.38
Gastroesophageal reflux symptoms, *n* (%)	5 (3.5%)	2 (4.3%)	0 (0.0%)	1 (6.7%)	0.73
Diarrhea, *n* (%)	10 (7.1%)	3 (6.5%)	0 (0.0%)	0 (0.0%)	0.91
Constipation, *n* (%)	10 (7.1%)	1 (2.2%)	1 (8.3%)	0 (0.0%)	0.46
Fecal incontinence, *n* (%)	0 (0.0%)	1 (2.2%)	0 (0.0%)	0 (0.0%)	0.34
Symptom complex					
Upper GI symptoms€, *n* (%)	12 (8.5%)	3 (6.5%)	0 (0.0%)	3 (20.0%)	0.32
Lower GI symptoms∲, *n* (%)	20 (14.2%)	5 (10.9%)	1 (8.3%)	0 (0.0%)	0.53

Data are presented as number (%).
€: A combined prevalence of early satiety, postprandial fullness, nausea, retching, vomiting, loss of appetite, abdominal fullness or bloating, gastroesophageal reflux symptoms, and dysphagia.
∲: A combined prevalence of diarrhea, constipation, and fecal incontinence.
The prevalence of GI symptoms across groups was compared using Chi-squared tests.
Note: the incidence of nausea, retching, vomiting was 0 in participants with diabetes, therefore, we did not compared these symptoms under different glucose-lowering therapies.

Table S4. Comparison of gastrointestinal symptoms in participants with diabetes under different HbA1c levels

	HbA1c < 6% (n=58)	6% ≤ HbA1c < 7% (n=93)	HbA1c ≥7% (n=63)	*P*
Total GI symptom, *n* (%)	8 (13.8%)	23 (24.7%)	12 (19.0%)	0.26
Abdominal pain, *n* (%)	1 (0.7%)	0 (0.0%)	1 (1.6%)	0.32
Irritable bowel syndrome, *n* (%)	0 (0.0%)	2 (2.2%)	2 (3.2%)	0.58
Ulcer-like dyspepsia, *n* (%)	0 (0.0%)	2 (2.2%)	0 (0.0%)	0.51
Early satiety, *n* (%)	3 (5.2%)	0 (0.0%)	2 (3.2%)	0.08
Postprandial fullness, *n* (%)	3 (5.2%)	2 (2.2%)	0 (0.0%)	0.13
Loss of appetite, *n* (%)	2 (3.4%)	0 (0.0%)	0 (0.0%)	0.07
Abdominal fullness or bloating, *n* (%)	2 (3.4%)	2 (2.2%)	1 (1.6%)	0.73
Gastroesophageal reflux symptoms, *n* (%)	2 (3.4%)	3 (3.2%)	3 (4.8%)	0.90
Diarrhea, *n* (%)	1 (1.7%)	8 (8.6%)	4 (6.3%)	0.24
Constipation, *n* (%)	2 (3.4%)	7 (7.5%)	3 (4.8%)	0.61
Fecal incontinence, *n* (%)	0 (0.0%)	1 (1.1%)	0（0.0%）	1.00
Symptom complex				
Upper GI symptoms€, *n* (%)	7 (12.1%)	5 (5.4%)	6 (9.5%)	0.32
Lower GI symptoms∲, *n* (%)	3 (5.2%)	16 (17.2%)	7 (11.1%)	0.09

Data are presented as number (%).

€: A combined prevalence of early satiety, postprandial fullness, nausea, retching, vomiting, loss of appetite, abdominal fullness or bloating, gastroesophageal reflux symptoms, and dysphagia.

∲: A combined prevalence of diarrhea, constipation, and fecal incontinence.

The prevalence of GI symptoms across groups was compared using Chi-squared tests.

Note: the incidence of nausea, retching, vomiting was 0 in participants with diabetes, therefore, we did not compared these symptoms under different HbA1c levels
